# Pioneering iPSC‐Based Therapies for Dementia: Enhancing Safety and Microenvironment Integration

**DOI:** 10.1002/alz70855_102827

**Published:** 2025-12-23

**Authors:** Sofia Gomes Lopes

**Affiliations:** ^1^ Polytechnic Institute of Setúbal (IPS), Almada, Setúbal, Portugal

## Abstract

**Background:**

Dementia is a significant global health challenge, affecting millions with profound cognitive and physical impairments. Despite advancements in palliative care, no curative therapies currently exist. Induced pluripotent stem cells (iPSCs) offer a promising solution to repair or replace damaged neurons. However, challenges such as immune rejection, integration into neural circuits, and long‐term viability remain critical barriers. This project addresses these obstacles by modifying the cerebral microenvironment, integrating advanced safety mechanisms, and employing biosensors for continuous monitoring.

**Objective:**

To develop a therapeutic strategy that combines iPSC‐based therapies with cerebral microenvironment modifications and advanced genetic engineering, ensuring safe and effective integration of iPSCs while reducing risks such as tumor formation and immune rejection.

**Methods:**

The therapeutic framework comprises three key components:

**Microenvironment Modification**:

Anti‐inflammatory interventions targeting microglia and astrocytes.

Reduction of oxidative stress through natural antioxidants like coenzyme Q10.

Cognitive stimulation, exercise, and tailored nutritional strategies to enhance neuroregeneration.

**iPSC Integration**:

To prevent teratoma formation, engineering iPSCs with CRISPR‐Cas9 to express tumor suppressor genes, such as p53 or RB1.

Integration of genetic circuits to trigger apoptosis in aberrant cells.

**Continuous Monitoring**:

Biosensors integrated with diagnostic systems for real‐time assessment of inflammation, immune responses, and other biomarkers, enabling early detection of complications.

**Results:**

Preliminary studies with in vitro models demonstrated that:

Modified iPSCs showed an 85% efficiency in differentiating into functional neurons.

Safety mechanisms effectively reduced aberrant cell proliferation by 90%.

Microenvironment interventions decreased inflammatory markers by 40% and improved cell viability in co‐culture models.

These findings provide evidence supporting the feasibility of this approach and its potential to overcome current limitations in iPSC‐based therapies.

**Conclusion:**

This project outlines a groundbreaking therapeutic strategy addressing key barriers in iPSC‐based dementia treatments, including immune rejection, tumor risk, and microenvironment compatibility. By integrating safety mechanisms, optimizing the cerebral environment, and employing biosensor monitoring, this approach holds promise for personalized neurodegenerative disease care, potentially restoring cognitive function and improving quality of life.

